# Modelling the initial stages of biocontrol of the invasive herb *Tradescantia fluminensis* by beetles

**DOI:** 10.1098/rsos.241939

**Published:** 2025-06-11

**Authors:** Vincent Lomas, Simone Cunha, Mingfeng Qiu, Dave Kelly, Alex James

**Affiliations:** ^1^ School of Mathematics and Statistics, University of Canterbury, Christchurch, New Zealand; ^2^ Ngāi Tahu Research Centre, University of Canterbury, Christchurch, New Zealand; ^3^ School of Biological Sciences, University of Canterbury New Zealand, Christchurch, New Zealand

**Keywords:** biocontrol, deterministic model, Monte Carlo sensitivity analysis

## Abstract

*Tradescantia fluminensis* is a herbaceous monocot originally from South America which is an invasive weed in many countries. Manual control is highly intensive and efforts have been made to establish colonies of three biocontrol beetles: *Neolema ogloblini*, *Neolema abbreviata* and *Lema basicostata*. Each beetle has its own characteristic life cycle and pattern of tradescantia damage. We construct a model of both plant and beetle population to assess the initial stages of interaction after the release of each biocontrol species. We parametrize the model using field data and give values for parameters which are hard to observe. We use the model to estimate the size of the released population needed for immediate plant control and test the sensitivity of this estimate to parameter estimates using a novel Monte Carlo method. Our results show that this may be a successful method of biocontrol under some circumstances, but the initial number of beetles released would be unrealistically large in some cases. We consider ways that this model could be expanded in future to evaluate longer-term interactions.

## 1. Introduction

*Tradescantia fluminensis* (Commelinaceae), a clonally spreading herb native to South America, is now established as a ground cover in native forests across New Zealand, Australia and southeastern parts of the United States. In its native range, *T. fluminensis* is kept in check by natural predators and typically has a mean dry biomass of just 164 g m^−^² [1]. However, in New Zealand, where it has become widespread in many lowland forests, the mean biomass can reach 455 g m^−^², with some areas showing biomass levels up to 1400 g m^−^² [2]. At these higher biomass levels, *T. fluminensis* creates a dense mat up to 60 cm deep that suppresses the growth of native seedlings and hinders forest regeneration. In New Zealand, *T. fluminensis* reproduces solely through vegetative means. A broken stem segment longer than 1 cm, especially if it contains a node (leaf with axillary bud), has a high chance of surviving and growing back [2]. Its rapid growth, absence of natural predators, and impact on native forest regeneration have led to its inclusion on New Zealand’s National Pest Plant Accord (2012), designating it as an unwanted organism under the NZ Biosecurity Act (1993).

Standish *et al.* [3] demonstrated that as *T. fluminensis* biomass increases, there is an exponential decline in the species richness and abundance of native seedlings. This decline is largely attributed to the reduction in light levels caused by *T. fluminensis*: beneath a full mat of *T. fluminensis* cover, light intensity drops to less than 1%. While newly germinated seedlings may be present under the *T. fluminensis* cover, they often fail to establish [2,3]. *Tradescantia fluminensis* is sensitive to light conditions; its biomass is strongly positively correlated with light intensity, with the highest densities observed at light levels ranging from 30 to 50% of open ground. At light levels above this range, although the biomass remains high, it does not correlate with light intensity [4].

Until 2012, control efforts for *T. fluminensis* in New Zealand relied on herbicide application or hand weeding. However, neither method has proven effective in achieving complete removal or preventing regrowth. As an alternative, artificial shading has been proposed to manage the plant [5]. The goal of shading is not to eradicate *T. fluminensis* but to lower its biomass to a level where native plant regrowth can occur. Once native woody seedlings grow to more than 30 cm, they can potentially shade the ground enough to suppress the *T. fluminensis*. More recently, biological control strategies have been explored in New Zealand, including the release of three species of chrysomelid beetles and a fungus [1].

The three beetle species *Neolema ogloblini*, *Neolema abbreviata* and *Lema basicostata* were released in New Zealand in 2011−2012 with the hope they would lower levels of tradescantia. They are all native to Brazil and Argentina. The beetles are closely related, similar in size, and adults of all three species feed on tradescantia leaves, but during the juvenile stage, they have very different feeding behaviours. *Neolema ogloblini*, the tradescantia leaf beetle, feeds on leaves during the juvenile period, decreasing the plant’s photosynthetic capacity. *Neolema abbreviata,* the tradescantia tip beetle, feeds on the plant tips during its juvenile period, damaging them and preventing further terminal growth on a particular stem. Juveniles of *L. basicostata,* the tradescantia stem beetle, bore through the stems, damaging the stems and associated nodes and leaves, and causing stem sections to break.

*Tradescantia fluminensis* has been the subject of numerous modelling approaches. Hogan & Myerscough [6] developed a spatial model using PDEs to investigate how the plant colonizes new areas by extension of stems rather than seed dispersal. James *et al.* [7] used a stochastic model to understand the growth of individual stems and calibrated the model to existing data on growth under different light conditions. Plank *et al.* [8] developed a dynamic model of the height of the dense mat formed by the plant and showed that under certain shade conditions growth can be limited.

Here, we build on the model of James *et al.* [7] and consider individual plant stems and the effect of different kinds of beetles. We start with a similar stochastic model but quickly switch to a mean-field model using ODEs that shows equivalent behaviour but is more analytically tractable. The model focuses on the initial stages of plant growth before environmental factors impose a carrying capacity, limiting the plant growth. We couple this model to a simple population model of beetles with two distinct life stages: juvenile and adult, and different grazing mechanisms for each beetle species. We parametrize our model, explicitly and implicitly, using data from numerous sources. The key model output is the initial size of the beetle population necessary to consume all the plants. Finally, we recognize the inexactness of our parameter estimations and use a novel approach for our sensitivity analysis: first, a Monte Carlo method to simulate the model at a wide range of parameter values around our original estimates and then a regression model to estimate the effect of each parameter on the required initial population size. Finally, we discuss how the current model could be expanded to include interactions once the plant and/or insects have reached carrying capacity.

## 2. Models

We start with a stochastic model of plant growth based on previous work [7] and similar to other plant-biocontrol models, e.g. Room [9]. We approximate this model with mean field differential equations (DEs). The plant model is parametrized using available data. We then present a simple DE model for a beetle population. The model is parametrized using available data for each species of biocontrol beetle. We then combine the two models to compare the effect of each type of beetle on initial plant growth.

### 2.1. Plant model

Our stochastic model uses a Poisson process that considers the four distinct parts of the plant: nodes, leaves and tips, connected by stems. This model is very similar to the one proposed by James *et al.* [7]. There are three distinct events that can occur:

(1) Any existing node can branch to form a new tip with probability $\lambda_{B}$. Nodes may branch more than once. Branched nodes keep their leaves.
(2) A tip can grow to form a new node with probability G. This new node will have a leaf.
(3) A node adjacent to a dead node can die with probability $\lambda_{D}$. Dead nodes do not have leaves.

**Figure 1.**
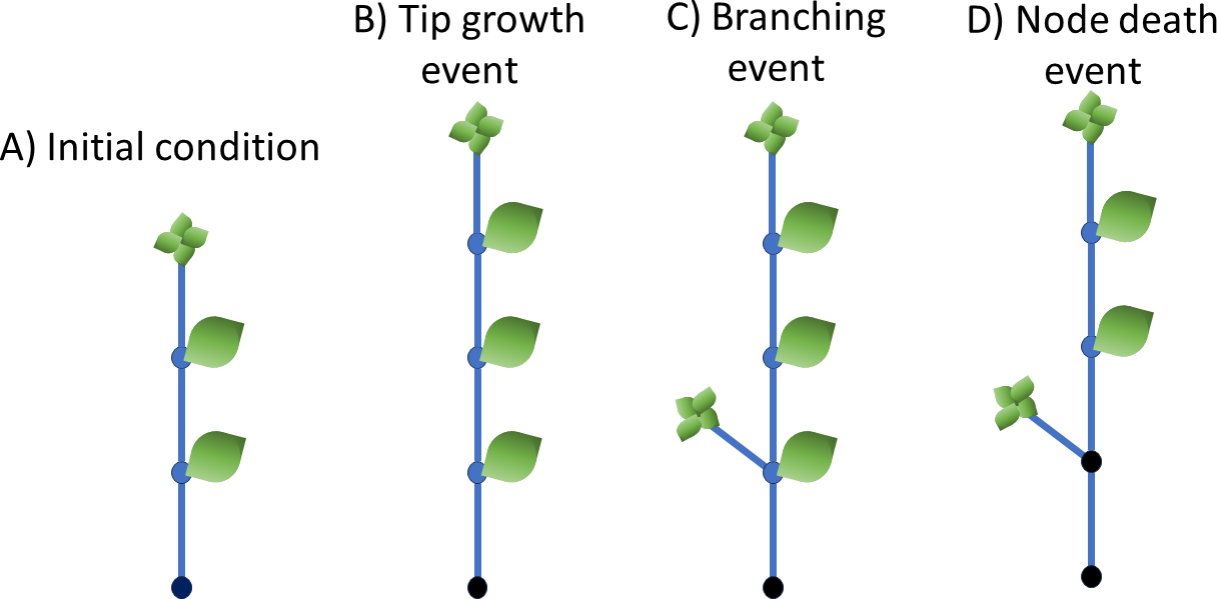
The plant growth model. (A) The initial condition of one dead node at the base (black), two live unbranched nodes and a tip. (B) A tip growth event creates a new live unbranched node. (C) A branching event creates a new tip. (D) A node death event kills the node and leaf adjacent to the dead node.

Figure 1 summarizes the plant growth model. The initial condition is a single, dead basal node with two living nodes and a single tip, connected as in figure 1. The model is realized with a constant time step dt=0.1. Parameter values with source references are given in table 1.

**Table 1.** Parameter values, descriptions, sources and references.

	description	param.	value	species	reference
plant growth	tip growth rate	$\lambda_{G}$	0.0948 day^−1^	*Tradescantia fluminensis*	[10]
	branching rate	$\lambda_{B}$	0.0027 day^−1^	*Tradescantia fluminensis*	[7]
	basal death rate	$\lambda_{D}$	0.0014 day^−1^	*Tradescantia fluminensis*	
beetle population	juvenile to adult transition rate	r	0.0238 day^−1^ (6 weeks juvenile lifespan)	*Neolema* *ogloblini* (leaf)	[11]
			0.0143 day^−1^ (10 weeks juvenile lifespan)	*Neolema abbreviata* (tip)	
			0.0130 day^−1^ (11 weeks juvenile lifespan)	*Lema* *basicostata* (stem)	
	birth rate	b	2.6786 day^−1^ (300 eggs in 16 weeks)	*Neolema* *ogloblini* (leaf)	[11]
			2.6786 day^−1^ (300 eggs in 16 weeks)	*Neolema abbreviata* (tip)	
			1.3393 day^−1^ (150 eggs in 16 weeks)	*Lema* *basicostata* (stem)	
	adult mortality rate	$\mu_{A}$	0.0089 day^−1^ (16 week adult lifespan)	all beetles	[11]
	juvenile mortality rate	$\mu_{J}$	3.561 day^−1^ (340% increase at 24 weeks)	*Neolema* *ogloblini* (leaf)	[12]
			2.267 day^−1^ (270% increase at 24 weeks)	*Neolema abbreviata* (tip)	[12]
			0.991 day^−1^ (300% increase at 24 weeks)	*Lema* *basicostata* (stem)	mean of previous two assumed.
plant–beetle interactions	adult grazing rate	$\gamma_{A}$	0.0661 day^−1^	all beetles	[10]
	juvenile grazing rate	$\gamma_{J}$	0.0661 day^−1^	*Neolema* *ogloblini* (leaf)	assumed (adult rate)
			0.0281 day^−1^	*Neolema abbreviata* (tip)	[10]
			0.0112 day^−1^	*Lema* *basicostata* (stem)	[10]
	adult (food) mortality	$\mu_{A2}$	$\mu_{A}=0.0089$ day^−1^	all beetles	assumed
	Holling II	α	10	all grazing	assumed

The stochastic model has a quasi-spatial element, as only nodes adjacent to a dead node can die. However, it is possible to make a simple, though not perfect mean field approximation. The number of tips and live nodes at time t are $T\left(t\right)$ and $N\left(t\right)$. New tips are created proportional to the number of nodes, and new nodes are created proportional to the number of tips. As death occurs through adjacency, node death is not proportional to the number of living nodes. Every time a branched node dies the death rate can increase as, after the new tip has grown, an additional node is adjacent to a dead node. We approximate this by assuming node mortality is proportional to the number of tips, an imperfect but hopefully good enough assumption. An alternative would be to use the number of branched nodes. However, this would be less accurate as this would decrease with node death, whereas tips live forever, so the mortality rate can only increase. This gives the set of DEs


$$\frac{\text{d}T}{\text{d}t}=\lambda_{B}N,$$

$$\frac{\text{d}N}{\text{d}t}=GT-\lambda_{D}T.$$

In this simple linear system, the plant will either live forever, growing exponentially with growth rate $\Lambda =\sqrt{\lambda_{B}\left(G-\lambda_{D}\right)}$, or it will eventually die completely if the growth rate is smaller than the death rate, i.e. $G<\lambda_{D}$.

In readiness for extending our model to include beetles, we introduce an additional variable for the number of leaves, $L\left(t\right)$. At this stage, every living node has a leaf giving


$$\frac{dL}{dt}=GT-\lambda_{D}T.$$

An immediate consequence of this model is that there is no carrying capacity, i.e. plant growth is exponential and unlimited. In reality growth of *T. fluminensis* is constrained by a wide variety of factors, including climate (it is susceptible to frost damage [13] and local environment (growth is limited by shade [8]). Although this is a model limitation, it allows us to focus on the initial phase of plant and beetle growth and estimate some parameters which are hard to observe. The implications of this model choice are discussed in §5.

### 2.2. Beetle model

In our simple model, beetles undergo two life stages—juvenile and adult. The juvenile stage covers several stages including egg and successive larval instars which are grouped together here [11]. Transition to the adult stage occurs as a Poisson process with rate r. Adults give birth to new juveniles at rate b throughout the whole adult stage. Adult and juvenile mortality are also Poisson processes with rates $\mu_{A}$ and $\mu_{J}$, respectively. Initially, we assume that the population is not food limited.

The adult population can be approximated by a mean-field system of DEs with $J\left(t\right)$ the number of juveniles and $A\left(t\right)$ the number of adults at time t,


$$\begin{array}{rl} \frac{dJ}{dt} & =-rJ+bA-\mu_{J}J\\ \frac{dA}{dt} & =rJ-\mu_{A}A. \end{array}$$

As with the plant model, this is a linear system and the dominant eigenvalue dictates the system’s growth or decay.

### 2.3. Plant–beetle interaction model

Our final model combines the plant and beetle dynamics. Here, beetle population growth is limited by food availability, and plant growth, i.e. food availability, is impacted by the beetle population. In their adult stage, all three beetle species feed on tradescantia leaves. During the juvenile stage they have different feeding behaviours.

To incorporate these feeding behaviours, we extend our plant model in the following ways:

(1) We introduce two additional variables, $L_{X}\left(t\right)$ and $T_{X}\left(t\right)$, for the number of leaves and tips which have been damaged by beetles, respectively.
(2) Node death, and correspondingly leaf death, is now proportional to the total number of tips both damaged and undamaged. This gives the extended plant equations, still in the absence of beetles, as

$$\begin{array}{rl} \frac{dT}{dt} & =\lambda_{B}N\\ \frac{dN}{dt} & =G\left(L\right)T-\lambda_{D}\left(T+T_{X}\right)\\ \frac{dL}{dt} & =G\left(L\right)T-\lambda_{D}\left(T+T_{X}\right)\\ \frac{dL_{X}}{dt} & =\lambda_{D}\left(T+T_{X}\right)\\ \frac{dT_{X}}{dt} & =0. \end{array}$$

(3) We assume that adult beetles feed on leaves at rate $\gamma_{A}$ which is the same for all three species of beetle. This grazing rate follows a Holling II process, i.e. grazing happens at full rate if the food source is widely available, but the rate decreases to zero when food availability becomes low. This modifies our two-leaf equations to be

$$\begin{array}{rl} \frac{\text{d}L}{\text{d}t} & =G\left(L\right)T-\lambda_{D}\left(T+T_{X}\right)-\gamma_{A}B\frac{L}{\alpha +L}\\ \frac{\text{d}L_{X}}{\text{d}t} & =\lambda_{D}\left(T+T_{X}\right)+\gamma_{A}B\frac{L}{\alpha +L}. \end{array}$$

(4) Depending on the species, juvenile beetles graze on tips at rate $\gamma_{JT}$, leaves at rate $\gamma_{JL}$ or nodes at rate $\gamma_{JN}$. If nodes are grazed then leaves will also be affected at the same rate. These grazing rates follow the same Holling II process as the adult grazing rate. Our final plant model is now

$$\begin{array}{rl} \frac{\text{d}T}{\text{d}t} & =\lambda_{B}N-\gamma_{JT}J\frac{T}{\alpha +T}\\ \frac{\text{d}N}{\text{d}t} & =G\left(L\right)T-\lambda_{D}\left(T+T_{X}\right)-\gamma_{JN}J\frac{N}{\alpha +N}\\ \frac{\text{d}L}{\text{d}t} & =G\left(L\right)T-\lambda_{D}\left(T+T_{X}\right)-\gamma_{A}B\frac{L}{\alpha +L}-J\frac{L}{\alpha +L}\left(\gamma_{JN}+\gamma_{JL}\right)\\ \frac{\text{d}L_{X}}{\text{d}t} & =\lambda_{D}\left(T+T_{X}\right)+\gamma_{A}B\frac{L}{\alpha +L}+J\frac{L}{\alpha +L}\left(\gamma_{JN}+\gamma_{JL}\right)\\ \frac{\text{d}T_{X}}{\text{d}t} & =\gamma_{JT}J\frac{T}{\alpha +T}. \end{array}$$

We must now extend the beetle model to account for the potential lack of food. This occurs in two ways:

(1) We assume that a lack of leaves results in an additional adult mortality mechanism. When leaf numbers are high mortality is unchanged at $\mu_{A}$ but as leaf numbers fall to zero, mortality increases by up to $\mu_{A2}$ through an additional Holling II term.
(2) Juvenile mortality is already high (egg laying rates are around 300 per adult lifetime), so we assume that an increase in mortality from lack of food will be negligible. Instead. we assume that, as adult beetles graze predominantly on leaves, a lack of leaves will affect the transition from juvenile to adult. We assume that when leaf numbers are low, this transition will be less successful, i.e. the juvenile population will continue to decrease at the same rate but the adult population will not increase accordingly. Again, this effect follows a Holling type II function: with high leaf numbers the transition is always successful, but as leaf numbers fall the probability of successfully transitioning falls, eventually to zero when the plant has no leaves.

In addition to these extensions, birth continues regardless of food availability. These assumptions result in the modified beetle model,


$$\begin{array}{rl} \frac{\text{d}A}{\text{d}t} & =rJ\frac{L}{1+L}-\mu_{A}A-\mu_{A2}A\left(1-\frac{1}{\alpha +L}\right)\\ \frac{\text{d}J}{\text{d}t} & =-rJ+bA-\mu_{J}J. \end{array}$$

Together with the plant equations we now have a system of seven ODEs.

## 3. Parameter estimates

While many of the model parameters can be taken directly from observations, some must be obtained indirectly by fitting model output to experimental observations. Our primary data sources for the plant parameters are two works that measured plant growth under different conditions. Cunha [10] conducted glasshouse experiments on plants with different combinations of biocontrol insects present and measured overall plant growth rate by leaf area. We assume the relative growth of leaf area in these experiments is directly related to the number of leaves. The control experiment used no biocontrol species. James *et al.* [7] measured plant growth in the field without biocontrol and estimated the probability that a node would branch and the probability of basal node death.

For the beetle model, there are a number of direct observations on all three species for birth rates and the length of the juvenile and adult stages. These are summarized by Landcare Research [11].

There are fewer direct measurements of plant growth with biocontrol. The most useful for this model is Cunha [10], who estimated the decrease in relative growth rate for each of the three biocontrol species. There are no direct estimates for the beetle–plant interaction parameters, instead we fit model output to these observations combined with some educated estimates for some parameters.

*Plant only model*. We use the estimates for basal death and branching rates, $\lambda_{D}$ and $\lambda_{B}$, given by James *et al.* [7], then use the relative growth rate (relative increase in leaf area) for the control plants in Cunha [10], Λ=0.016 day^−1^, to estimate the growth rate, $\lambda_{G}$, by equating the relative growth rate with the largest eigenvalue of the plant model,


$$\Lambda =\sqrt{\lambda_{B}\left(G-\lambda_{D}\right)}.$$

All parameter values are given in table 1.

*Beetle-only model*. All three species of beetle have an expected adult lifespan in captivity of four months [11], which is used to estimate the intrinsic adult mortality rate $\mu_{A}$. The expected time from egg laying to adulthood is different for each species of beetle [11] and can be used to estimate the three values of the transition rate from juvenile to adult, r. Similarly, we use the estimated number of eggs laid in a lifetime [11] by each species to find the three values of b. The final parameter in the beetle model is $\mu_{J}$, the mortality rate of juveniles. There are no available data for this, so instead we use data from van der Walt [12]. In a controlled experiment populations were raised in glasshouse experiments with abundant food. The population increase after 24 weeks was recorded. By comparing this with the expected population growth rate from the largest eigenvalue in our beetle-only model, we can estimate juvenile mortality.

*Beetle–plant model*. For the interaction model, parameter estimates are more difficult. Our approach is to make assumptions for the less crucial parameters, testing these assumptions later in the sensitivity analysis, and then use available data to estimate the more important parameters. The four additional parameters are: the adult and juvenile grazing rates, $\gamma_{A}$ and $\gamma_{J}$; the Holling II parameter, α; and the adult food-related mortality rate, $\mu_{A2}$.

Our first assumption is that the adult grazing rates and the adult mortalities are the same for all three species of adult beetle, since adults of all three species graze predominantly on leaves, and they are of similar size. We assume these similarities will be seen in the corresponding parameters. The juvenile grazing rate is very different, as juveniles of each species feed on a different part of the plant. We assume the juvenile grazing rates are different for each species.

The Holling II parameter is seen in all the grazing rates for stems, leaves and tips. This parameter controls when grazing is proportional to the amount of food resource available and when food is abundant and simply proportional to the number of grazers. We start with a value of 1, which means it is only at very low food availability levels that food becomes a limiting factor. As all three species are mobile for most of their juvenile period, this corresponds to individuals being able to move to an alternative leaf, node or stem when one has been eaten if another is available. Grazing is only food limited when almost all leaves (or nodes or stems) have been consumed. Adult mortality due to lack of leaves is also regulated by the same Holling II parameter and the additional mortality, $\mu_{A2}$. With no data to estimate this, we assume that when the food supply is very limited, the adult mortality rate can double from its usual rate, i.e. $\mu_{A2}=\mu_{A}$.

To estimate the adult grazing rate, we compare the model output of the leaf-eater model with the decrease in relative leaf area observed by Cunha [10]. At this stage, our interaction model still has two unknown parameters, the adult and juvenile grazing rates. As we have only one observation to fit to, we make the assumption that the juvenile grazing rate is the same as the adult grazing rate, i.e. $\gamma_{JL}=\gamma_{A}$. This allows us to numerically fit the predicted model growth rate from the first eight weeks of growth (the length of the experiments) to the relative leaf growth rate seen in the *N. ogloblini* (leaf-eating) beetle experiments (Table 3-3 in [10]).

Once these parameters, in particular the adult grazing rate, which is the same for all three species, have been determined, we can estimate the final two parameters: the juvenile grazing rates for *L. basicostata* (stem-eating) and *N. abbreviata* (tip-eating) beetles. These are estimated in the same way as the grazing rates for leaf eaters, i.e. by fitting the first eight weeks of model-predicted growth to the decrease in plant growth rate for the *N. abbreviata* (tip-eating) and *L. basicostata* (stem-eating) beetles observed by Cunha [10].

## 4. Results

**Figure 2.**
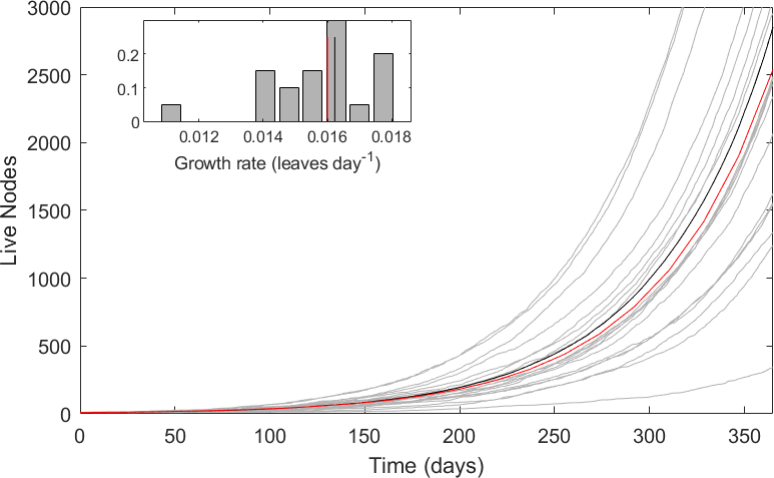
Plant growth in the plant-only model. Main panel: live nodes. Grey lines—20 individual stochastic simulations. Black line—mean of 1000 stochastic runs. Red line—solution of full DE model. Inset: the distribution of the stochastic growth rates and the mean growth rates (colours as previously).

Figure 2 shows example results of 20 simulations (grey lines) with a time step of 0.1 days, the mean value of the stochastic process (black line from 1000 simulations) and the DE results (red line) at the parameter values of table 1. The DE is a good approximation to the stochastic process at these parameter values.

The combined plant–beetle model shows two distinct types of behaviour depending on the initial conditions. If the initial beetle population is small compared with the number of plant leaves, the plant and beetle will coexist, and both the plant and beetle population will grow exponentially, though the plant will grow slower than would have been expected in the absence of beetles. In this case, we measure the damage caused by the beetle on the plant by comparing the number of leaves after 1000 days with the number expected with no control. If the initial population of beetles is large compared with the number of leaves, the beetles will consume all the available leaves. In our simple model, the beetle population will then die from starvation. In reality, this represents a successful outcome because, in the field, we would expect the beetle population to move on to an area with more available food and continue to damage the plant. We define one key model output as the smallest initial beetle population release size which results in all leaves being consumed. This represents initial heavy damage to the plant, and is proportional to the per-beetle impact on the (constant) initial number of leaves. Under these conditions, if the beetle population is maintained (in a field situation by feeding on adjacent tradescantia), plant growth should be suppressed, representing successful biocontrol.

**Figure 3.**
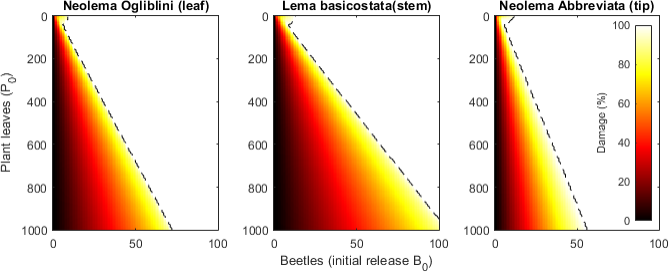
The effect of the beetles on the plant depends on the size of the initial release. Colour shows the amount of leaf damage relative to no beetles for different-sized plants and number of beetles released after 1000 days. For each species, the model predicts a critical release size needed to consume all the leaves (dashed line).

We run the model for each of the three beetle species at a range of different initial conditions. In each case, the initial conditions, at time t=0, for the plant are to have $P_{0}$ nodes, $N\left(0\right)=P_{0}$, 10 nodes for each tip, $T\left(0\right)=P_{0}/10$, and one leaf on each node, $L\left(0\right)=P_{0}$. There are no damaged leaves or tips, $L_{X}\left(0\right)=T_{X}\left(0\right)=0$. The initial beetle population is $B_{0}$ adults and no juveniles. Figure 3 shows damage caused by the beetles, i.e. the relative reduction in the number of leaves after 1000 days, for a range of initial conditions. The threshold for all leaves being consumed is approximately a linear function of beetles released to the plant leaves. For *N. ogliblini* and *N. abbreviata* at least eight adult beetles per 100 plant leaves are needed to destroy the plant. *Lema basicostata* needs slightly more at approximately 11 beetles per 100 leaves.

## 5. Model sensitivity

We test the model sensitivity to the various parameters using a Monte Carlo approach. We run 5000 simulations each at a different, randomly chosen, parameter. Each parameter is scaled by a random variable chosen from a uniform distribution, independently of the other parameter values in that particular simulation,


$$\bar{{param}_{i}}=X_{i}{param}_{i}\;\text{where}\;\;X_{i}∼U\left(0.5,2\right)\;\;\text{and}\;\;i=1...\text{number of parameters.}$$

This full independence across the parameter set allows one simulation to be run with, for example, high plant growth, low beetle mortality and high grazing, and then another simulation to have medium plant growth, high mortality and low grazing. For each simulation, we calculate the minimum number of beetles per 100 plant leaves that need to be released to ensure all leaves are eaten.

We run the Monte Carlo sensitivity simulations 5000 times for each of the three types of beetle. We then use a linear regression model to test the size of the effect of each parameter on the model outputs. As the parameters are all scaled, the effect size for each parameter is the coefficient in the linear model,


$$B_{0}\;∼\sum \beta_{i}X_{i}.$$

As the number of simulations that can be run is unlimited, the significance of the coefficients is uninformative—with enough simulations, all the coefficients will be significant! However, the coefficient value is stable to the number of simulations, and coefficient values for each parameter are comparable as only the scaling factor is included in the linear model, which has the same range (0.5,2) for all parameters. This allows us to estimate the relative impact of various plant and insect parameters on the immediate impact of the biocontrol agents. A positive coefficient implies that an increase in that parameter requires more beetles to be released to reach the critical threshold. A large negative coefficient means that fewer beetles would be required.

**Figure 4.**
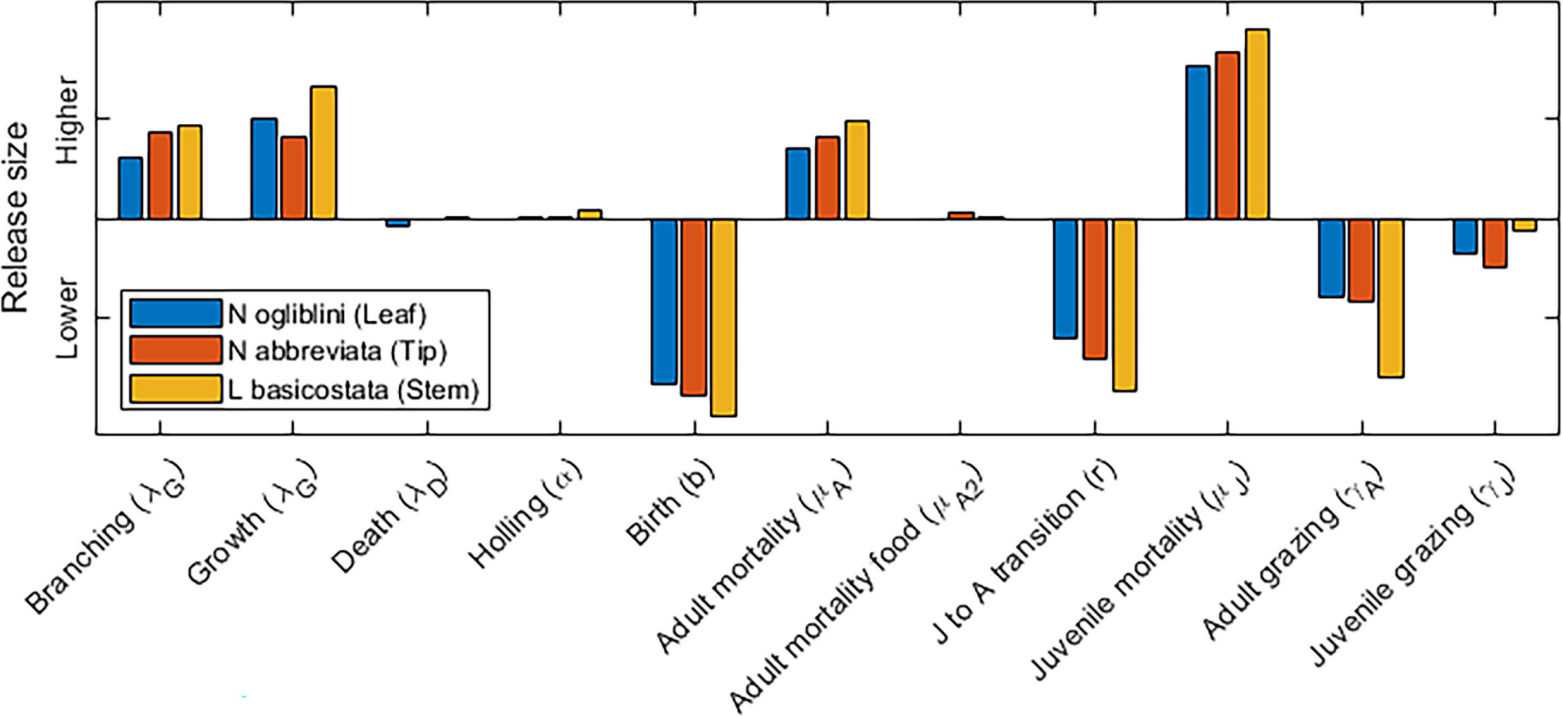
The effect of increasing model parameters on the critical release size necessary to consume all leaves. Positive coefficients imply that an increase in that parameter will result in a higher population size needed to reach the critical threshold.

Figure 4 shows the effect size in the linear model for each parameter value. One of the biggest factors affecting biocontrol is the plant growth rate; plants that grow faster and branch more frequently will require more beetles to be released. Correspondingly, environments which are not ideal for the beetle population, resulting in, for example, lower birth rates, higher adult or juvenile mortality will also need larger populations to be released. These effects are almost always strongest for the *L. basicostata* beetles. At the parameter values chosen, the model is relatively unaffected by the choice of the model-specific parameters like the Holling parameter.

In addition to parameter sensitivity, models are sensitive to model choice. As our model focused on the initial growth stages, it did not include terms for carrying capacity. This could be done by altering the first term of the tip growth equation to


$$\frac{\text{d}T}{\text{d}t}=\lambda_{B}N\left(1-\frac{N}{K}\right)-\gamma_{JT}J\frac{T}{\alpha +T}.$$

An immediate consequence of this would be that, in the cases where our model predicts plant survival and exponential growth of both the plant and the beetle population, the plant would reach carrying capacity and stop growing. The time taken to achieve this would depend on the size of the carrying capacity, K, and the net growth rate after beetle damage. Brief explorations of this extended system show that as plant growth slows, the beetle population would continue to increase and, in many cases, cause enough damage to kill the plant. However, these stages of plant and beetle growth are heavily dependent on other environmental factors which affect population growth rates, including temperature, drought, flooding, seasonal changes and predation by natural enemies on the beetles. To be useful, a model would need to include the most important of these to understand the longer-term consequences of the plant and beetle populations coexisting, or the level of longer-term biocontrol.

## 6. Discussion

We have presented a relatively simple model for the initial stages of tradescantia growth and the subsequent interactions with three species of biocontrol beetles. The model was parametrized with available data, and an extensive sensitivity analysis using Monte Carlo simulations was performed. The simple nature of the model allows for two outcomes: complete consumption of all leaves (which in the model results in the death of both the plant and the beetle) or coexistence. We interpret the first solution as a victory for the beetle: that particular patch of plant has been quickly killed, and the beetle population, which is relatively mobile, can move on to other nearby plants before it dies of starvation. Extinction of the beetles in our models is an artefact of the closed system, where beetles were unable to emigrate to find more food having defoliated the model plants. Successful biocontrol agents often defoliate patches of their plant hosts, but can then move on to new patches (many biocontrol insects, including these three beetles, can fly). In cases of successful weed biocontrol, for example, control in many countries of St Johns Wort (*Hypericum perforatum*) by another group of chrysomelid beetles, principally *Chrysolina hyperici* and *Chrysolina quadrigemina* [14–17], the host plant can be widespread but at low densities, and the biocontrol agents disperse to find patches of the plant and defoliate them. In the tradescantia system, forest patches with suitable habitat often have extensive stands of the plant, so that beetle local extinction through total defoliation at the site would seem unlikely.

In reality, the situation is not so clear cut as our model. If a released population quickly consumes all leaves on a plant (as is often seen in confined glasshouse experiments [12]), it is not a certainty that the beetle population would successfully move to another plant and continue the trend there. Again, it would depend on the distance to the next patch of plants, the mortality rate of beetles during emigration, and the ratio of beetles to plant leaves at the new site. Also, some of the initial densities required in the model for complete defoliation are higher than could realistically be achieved in the field. Although it is well known that biocontrol agent establishment becomes more likely with increasing numbers of individual agents released [18] that is a separate effect, driven by factors like mate finding and agent dispersal at very low densities. However, there are previous reports which demonstrate the principle that boosting biocontrol agents initially to high densities might allow them to overwhelm the host plant. In Papua New Guinea, adding nitrogen fertilizer to patches of *Salvinia molesta* allowed the biocontrol beetle *Cyrtobagous salviniae* to increase in numbers, at which point beetle damage caused plant nitrogen concentrations to increase, allowing the beetle to increase faster than the plant and achieve control [19].

Secondly, the defoliated plants at the original site will not necessarily die [2], and if the plants resprout then beetles would have to find them again for long-term control. There are important differences between observed low impacts in the field and in our models. Lack of beetle persistence in the field can be caused by many factors, including insufficiently large founder beetle populations (perhaps interacting with lack of aggregation behaviour by beetle individuals at low densities), unfavourable climate at the novel release site, inclement weather events (the beetles are susceptible to drowning during floods) or natural enemies of the beetles such as parasitoids. None of those factors could be included in our models, or in the glasshouse experiments of Cunha [10]. However, these models can suggest which parts of the life cycle have the largest effects on prospects of control and the relative impact of the three different beetles on tradescantia.

The coexistence solution predicted here could also, in reality, still be a positive outcome because both plant and beetle populations grow exponentially. The plant initially has a higher growth rate (otherwise the beetle would have eaten all the leaves), so in our model, the plant keeps ahead of the beetle. However, in reality, the plant would soon reach carrying capacity and its growth would decrease to zero, potentially allowing the beetle to catch up. The outcome then depends on a number of factors not in our current models, including the carrying capacity of the plant, and factors limiting or preventing beetle growth (such as predation by natural enemies, and suitability of local environmental conditions). To fully understand these longer-term outcomes of biocontrol, the plant model would need to be modified to include a plant carrying capacity based on environmental factors. This may include seasonal temperature and rainfall changes which would also have an effect on the beetle population growth rates. A model extension of this sort would be useful to understand the wide range of outcomes seen from tradescantia beetle releases across New Zealand. In the North Island, beetles are establishing well at some sites and may be starting to reduce tradescantia biomass [20]. Conversely, in the cooler climate of the South Island, Cunha [10] found little evidence of leaf damage at numerous beetle release sites and beetles were only resighted at 1 of 10 sites 2 years after their release. That model extension will be easier based on our demonstration that the DE equations adequately represent the stochastic system outcomes, and our estimation of several parameters in this system that are hard to observe.

## Data Availability

This article uses previously published data, full references are given for each case. We have also uploaded code that when run should create the figures from the paper and calculate all the derived parameter values. Supplementary material is available online [21].
